# Reducing Solvent Consumption in Reductive Catalytic Fractionation through Lignin Oil Recycling

**DOI:** 10.1021/acssuschemeng.4c04089

**Published:** 2024-08-14

**Authors:** Jun Hee Jang, Júlia Callejón Álvarez, Quinn S. Neuendorf, Yuriy Román-Leshkov, Gregg T. Beckham

**Affiliations:** † Renewable Resources and Enabling Sciences Center, National Renewable Energy Laboratory, Golden, Colorado 80401, United States; ‡ Center for Bioenergy Innovation, Oak Ridge National Laboratory, Oak Ridge, Tennessee 37830, United States; § Department of Chemical and Biological Engineering, University of Colorado Boulder, Boulder, Colorado 80303, United States; ∥ Department of Chemical Engineering, Massachusetts Institute of Technology, Cambridge, Massachusetts 02139, United States

**Keywords:** reductive catalytic fractionation, lignocellulosic biomass, lignin valorization, process intensification, solvent reduction, reaction engineering

## Abstract

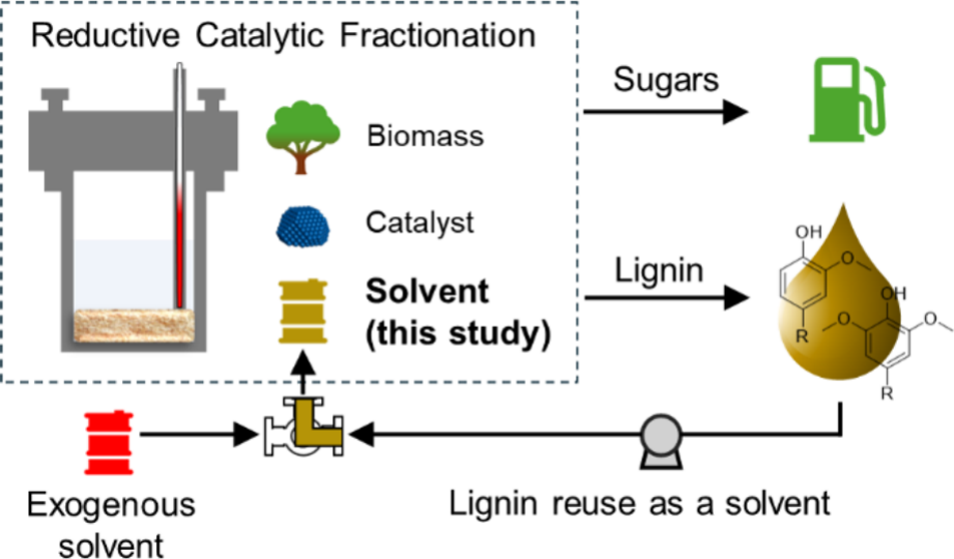

Reductive catalytic fractionation (RCF) enables the simultaneous valorization of lignin and carbohydrates in lignocellulosic biomass through solvent-based lignin extraction, followed by depolymerization and catalytic stabilization of the extracted lignin. Process modeling has shown that the use of exogenous organic solvent in RCF is a challenge for economic and environmental feasibility, and previous works proposed that lignin oil, a mixture of lignin-derived monomers and oligomers produced by RCF, can be used as a cosolvent in RCF. Here, we further explore the potential of RCF solvent recycling with lignin oil, extending the feasible lignin oil concentration in the solvent to 100 wt %, relative to the previously demonstrated 0–19 wt % range. Solvents containing up to 80 wt % lignin oil exhibited 83–93% delignification, comparable to 83% delignification with a methanol–water mixture, and notably, using lignin oil solely as a solvent achieved 67% delignification in the absence of water. In additional experiments, applying the RCF solvent recycling approach to ten consecutive RCF reactions resulted in a final lignin oil concentration of 11 wt %, without detrimental impacts on lignin extraction, lignin oil molar mass distribution, aromatic monomer selectivity, and cellulose retention. Overall, this work further demonstrates the potential for using lignin oil as an effective cosolvent in RCF, which can reduce the burden on downstream solvent recovery.

## Abstract 2

This study demonstrates the effectiveness and stability of lignin oil as a cosolvent in reductive catalytic fractionation processes

## Introduction

Reductive catalytic fractionation (RCF) is a lignin-first biorefinery method, in which lignin is extracted from intact biomass through the use of a polar protic solvent and catalytically stabilized, in the presence of a hydrogen source, into a lignin oil rich in aromatic monomers and C–C linked oligomers.^1−9^ Recent techno-economic analysis (TEA) and life cycle assessment (LCA) have suggested that the implementation of RCF at an industrial scale faces challenges concerning both costs and environmental impacts, with one of the major contributors being the use and recycling of exogenous organic solvent.^10−12^

Toward the reduction or elimination of exogenous organic solvent usage, several groups have demonstrated that lignin oil, a mixture of lignin-derived aromatic monomers and C–C linked oligomers produced by RCF, can be used as a cosolvent with alcohol or alcohol–water mixtures without negatively affecting RCF performance.^12−14^ In previous work, we used a flow-through RCF reactor configuration to demonstrate the concept of a multipass strategy, wherein the RCF effluent is recycled and used in subsequent flow-through RCF steps without intermediate lignin oil recovery. This approach enabled a reduction in the overall solvent-to-biomass ratio from 48 to 1.9 L/kg, which is below the limit of the feasible solvent loading in a single-pass batch reaction of ∼4 L/kg.^14^ Notably, the multipass approach did not sacrifice fractionation efficiency nor lignin oil quality when the solvent contained up to 12 wt % lignin oil. Subsequently, Arts et al. reported that recycled RCF effluent with simulated solvent compositions comprising methanol, methyl acetate, acetic acid, water, and lignin oil (0–19 wt %) enhanced lignin extraction compared to methanol-based RCF.^12^ The presence of water and acid in the simulated solvent mixture also led to comparable monomer yields and hemicellulose coextraction. The recycling of the product and solvent mixture was also applied for solubilization of birch bark by Kumaniaev et al., reducing the solvent-to-biomass ratio from 10 to 3.3 L/kg through three consecutive recycles.^13^

Building on these previous findings,^12−14^ this study aimed to investigate the effects of incorporating varying concentrations of lignin oil (0–100 wt %) in the solvent for RCF, thus significantly extending the ranges previously tested. We first conducted RCF experiments in batch reactions with different lignin oil concentrations in methanol–water solvent mixtures and compared their fractionation efficiency (83–93% delignification with solvent mixtures containing up to 80 wt % lignin oil). Subsequently, we also performed ten successive fractionation cycles, recycling the RCF effluent and accumulating lignin oil up to 11 wt % in the solvent and measured fractionation efficiency (51–68% delignification). Overall, this work further demonstrates that the RCF processes can viably operate with reduced reliance on exogenous organic solvents.

## Results and Discussion

### RCF with Lignin Oil as a Cosolvent

**Figure 1:**
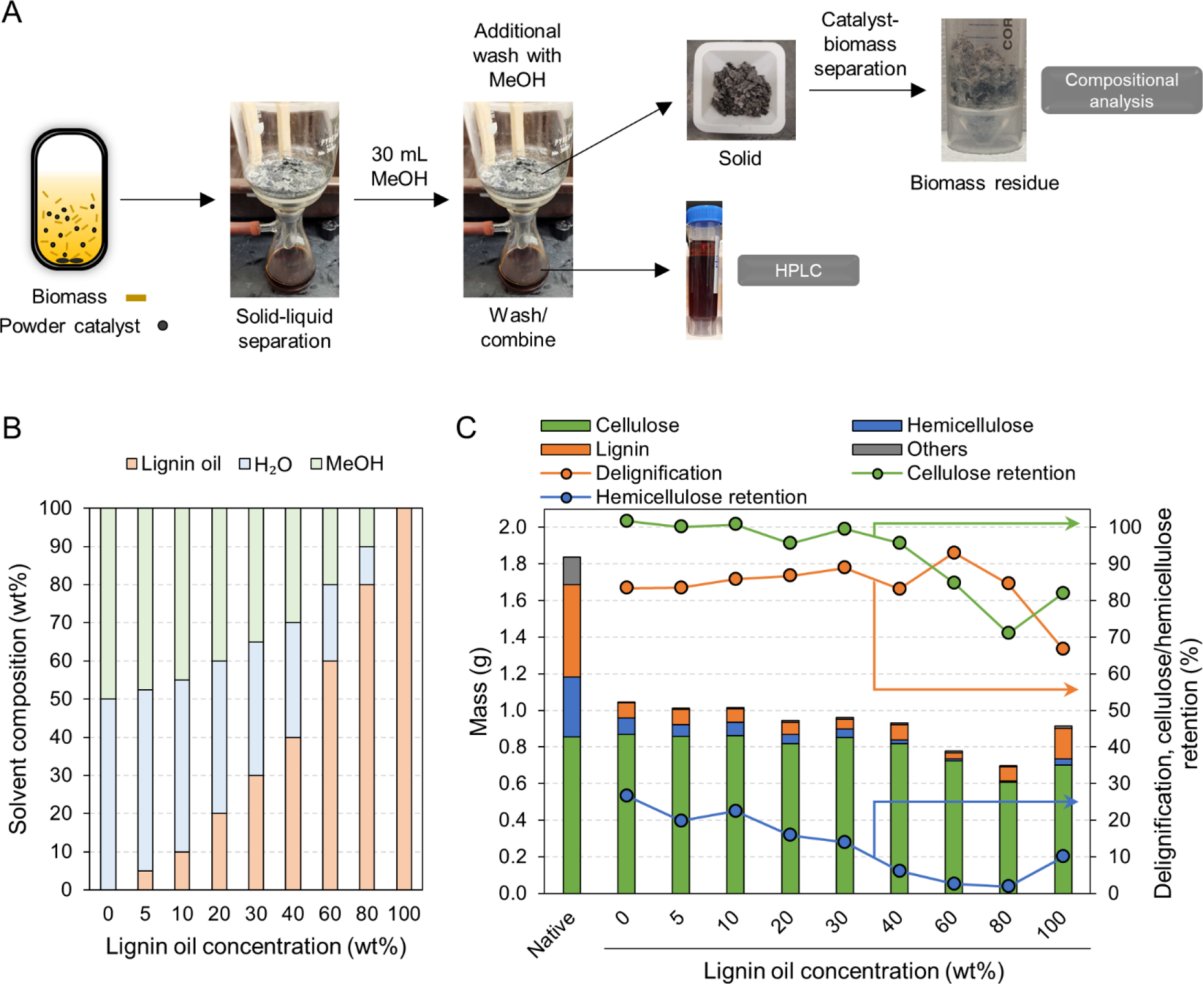
(A) The experimental scheme of RCF reactions with varied solvent compositions and the analyses of liquid and solid streams. (B) Solvent compositions with lignin oil concentrations varying from 0 to 100 wt %. The total solvent amount was maintained at 18 g by supplementing with a 1:1 w/w methanol–water mixture. (C) Compositional analysis of native and post-RCF pulp samples obtained using different solvent compositions. Delignification and the cellulose/hemicellulose retention were calculated based on the compositional analysis data. The secondary *x*-axis label indicates the concentration of lignin oil in the solvent for each RCF reaction. Others include ash, extractives, acetyl, and free sugars. RCF reaction conditions: 75 mL batch reactor, 2 g of hybrid poplar, 18 g of solvent, 400 mg of 5 wt % Ru/C, 200 °C, 30 bar H_2_ (at room temperature), 3 h (after 0.5 h heating ramp). Table S1 contains the quantitative information for the data shown here.

To evaluate the impact of lignin oil as a cosolvent on RCF fractionation efficiency, RCF reactions with varied solvent compositions were conducted in a 75 mL batch reactor by adding 2 g of hybrid poplar (26 wt % lignin content), 18 g of solvent (9 g solvent/g biomass), 400 mg of 5 wt % Ru/C, and 30 bar of H_2_. We used 20% catalyst loading to avoid catalyst-limited conditions since lignin oil included in the solvent could compete for adsorption sites on the catalyst surface with lignin oil extracted from the biomass during RCF.^14^ The reactor vessel was heated to 200 °C for 30 min and then maintained at that temperature for 3 h. The reaction mixture was subsequently separated and analyzed to investigate the fractionation efficiency (Figure 1A). The solvent systems included eight solvent mixtures that varied the lignin oil concentration from 0 to 100 wt %, each blended with a 1:1 w/w methanol–water mixture to maintain 18 g of each solvent mixture (Figure 1B). To vary feed concentrations, poplar lignin oil was prepared through a 3 L scale RCF reaction with methanol and a 5 wt % Ru/C catalyst. The produced RCF oil was then subjected to liquid–liquid extraction and Schlenk drying under vacuum to separate the lignin oil from soluble sugars.^15^ Different solvent compositions were formulated by adjusting lignin oil concentrations in the solvent mixture.

Batch RCF reactions at 200 °C for 3 h with a 1:1 w/w methanol–water mixture extracted both lignin and hemicellulose, with a delignification extent of 83% and hemicellulose retention of 27%, leaving cellulose intact in the pulp with a retention of 102% (Figure 1C), consistent with previous studies.^16−18^ When the lignin oil concentration increased up to 40 wt % in the solvent mixture, the levels of delignification (83–89%) and cellulose retention (95–102%) were preserved (Figure 1C and Table S1). These results are in line with previous studies by Jang et al. and Arts et al., which reported no negative impact on fractionation efficiency with up to 12 and 19 wt % lignin oil in the solvent, respectively.^12,14^ Moreover, solvents with 60 and 80 wt % lignin oil exhibited 93 and 85% delignification extents, respectively, demonstrating the effectiveness of lignin oil-enriched methanol–water solvents in lignin extraction. These results show an increase of the range of viable oil concentrations to 80 wt %, surpassing the previously tested concentrations of 0–19 wt %. A reaction using lignin oil alone as a solvent (100 wt % in Figure 1) achieved a delignification extent of 67%, comparable to previously reported delignification values from RCF with methanol or ethanol (50–70%).^16,18−22^ This demonstrates the ability of RCF-driven lignin oil to act as a solvent for RCF processes, likely due to the presence of phenolic and aliphatic hydroxyl groups in lignin oil. Similarly, Kim et al., used lignin oil, prepared by hydrocracking lignin pyrolysis oil, as a solvent for the hydrodeoxygenation of lignin pyrolysis oil without relying on exogenous solvents.^23^

The addition of lignin oil in the solvent led to carbohydrate extraction. As the concentration of lignin oil in the solvent increased up to 40 wt %, hemicellulose retention gradually decreased from 27 to 6%, while cellulose retention remained higher than 95%. Reactions using solvent systems enriched with 60–80 wt % lignin oil not only led to reduced hemicellulose retention down to 2% but also notable reductions in cellulose retention, down to 71% at 80 wt % lignin oil concentration. Compared to the reaction with 80 wt % lignin oil, the reaction with 100 wt % lignin oil exhibited less carbohydrate extraction with cellulose and hemicellulose retention at 82 and 10%, respectively. We posited that the noticeable increase in carbohydrate extraction with lignin-rich solvents may be a result of acidic components, such as acetic acid, present in the lignin oil. However, analyses using high-performance liquid chromatography (HPLC) and gas chromatography (GC) revealed that the prepared lignin oil contained no acetic acid or methyl acetate (Table S2), which might have been present in the initial oil obtained from RCF, but that were presumably removed during the liquid–liquid extraction and Schlenk drying process. The reaction effluent from the 60 wt % lignin oil solvent showed similar levels of acetic acid and methyl acetate relative to those extracted from the biomass substrate during RCF with methanol–water (0 wt % solvent). This finding excludes the possibility that the buildup of acidic components led to lower polysaccharide retention extents, instead suggesting that the lignin oil itself or high concentrations of lignin oil in methanol–water promoted carbohydrate extraction. One possibility is that acid environment formed by phenolic protons in lignin oil contributed to the carbohydrate extraction. Compared to lignin oil only (100 wt %), the presence of methanol and water (80 wt % lignin oil) as nucleophiles may facilitate the acid-catalyzed carbohydrate cleavage.

**Figure 2:**
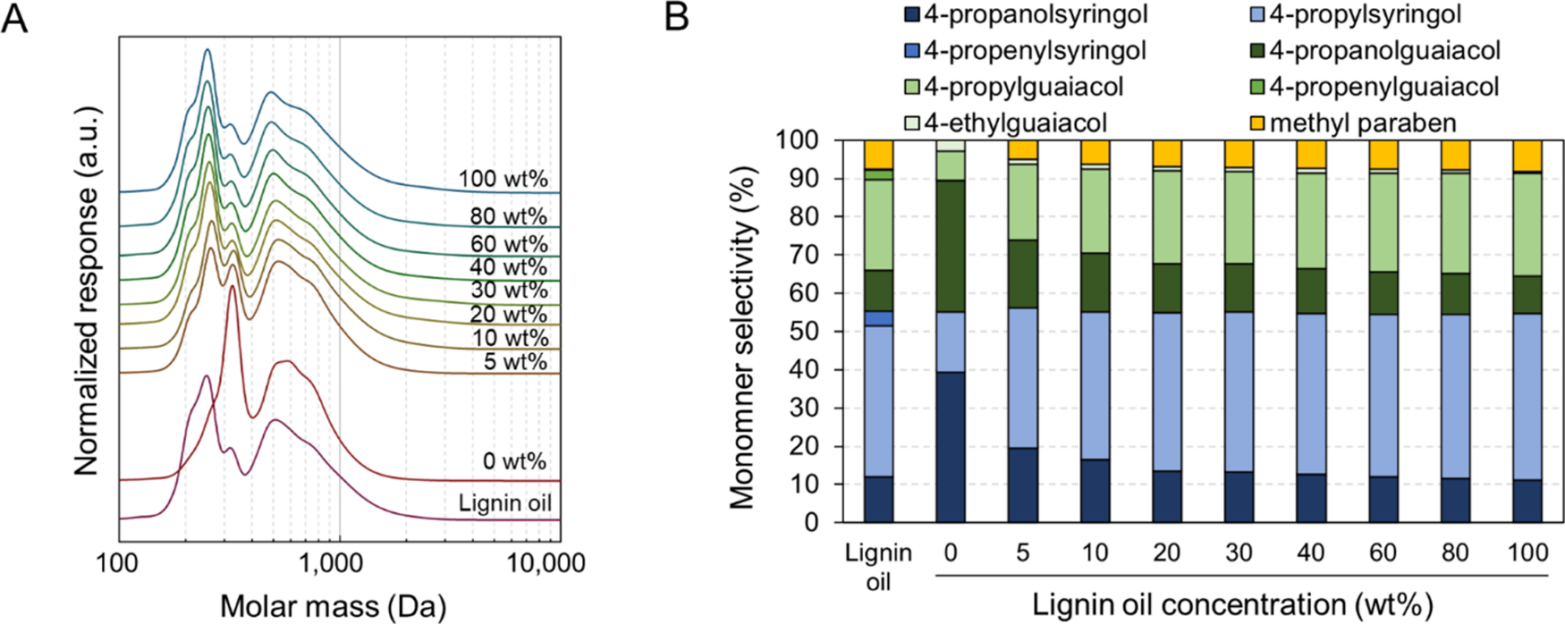
Characterization of the as-prepared lignin oil (labeled “Lignin oil”) and lignin oils obtained post-RCF. After RCF, the lignin oil was separated from soluble sugars through liquid–liquid extraction. (A) GPC traces after acetylation and (B) monomer selectivity. Table S3 contains the quantitative information for the data shown here.

The molar mass and monomer distributions of lignin oils obtained from each reaction are compared in Figure 2. Lignin oil produced in methanol (denoted as “Lignin oil” in Figure 2) exhibited clear peaks at 260, 320, and 500 Da, representing monomers, dimers, and trimers, respectively. The monomer fraction featured 4-propylguaiacol and 4-propylsyringol as the predominant monomers, with propanol and propenyl-substituted monomers present in small quantities. In reactions wherein RCF was conducted with a methanol–water mixture without incorporating lignin oil as part of the solvent, the produced lignin oil showed a reduced monomer peak relative to the lignin oil produced in methanol, primarily consisting of 4-propanolguaiacol and 4-propanolsyringol. Similarly, Renders et al. observed propanol monomers as the major RCF lignin monomer products from RCF at 200 °C using *n*-butanol–water solvent and Ru/C catalyst.^24^ Several RCF studies, however, primarily produced propyl monomers in a methanol–water mixture.^16,18^ This discrepancy arose depending on the catalyst, solvent, reaction conditions, and reactor system.^18,24^ The significant reduction of the monomer peak was due to condensation occurring in the presence of water,^18^ leading to an increased intensity of dimer peak (Figure 2A). As the lignin oil concentration in the solvent increased, while the quantity of lignin oil extracted from the biomass remained relatively constant, the molar mass and monomer distributions of the resulting lignin oil approached that of the lignin oil used as the solvent. Conducting RCF with a solvent composed entirely of 100 wt % lignin oil led to the saturation of minor propenyl monomers, probably corresponding to the reduced intensity of a bump at 210 Da in the gel permeation chromatography (GPC) trace of the lignin oil. The overall molar mass and monomer distributions, however, did not change noticeably, demonstrating the stability of lignin monomers and oligomers under the reaction conditions and suggesting the reusability of lignin oil as a solvent.

### Subsequent Ten Fractionation RCF with Recycling Lignin and Solvent

**Figure 3:**
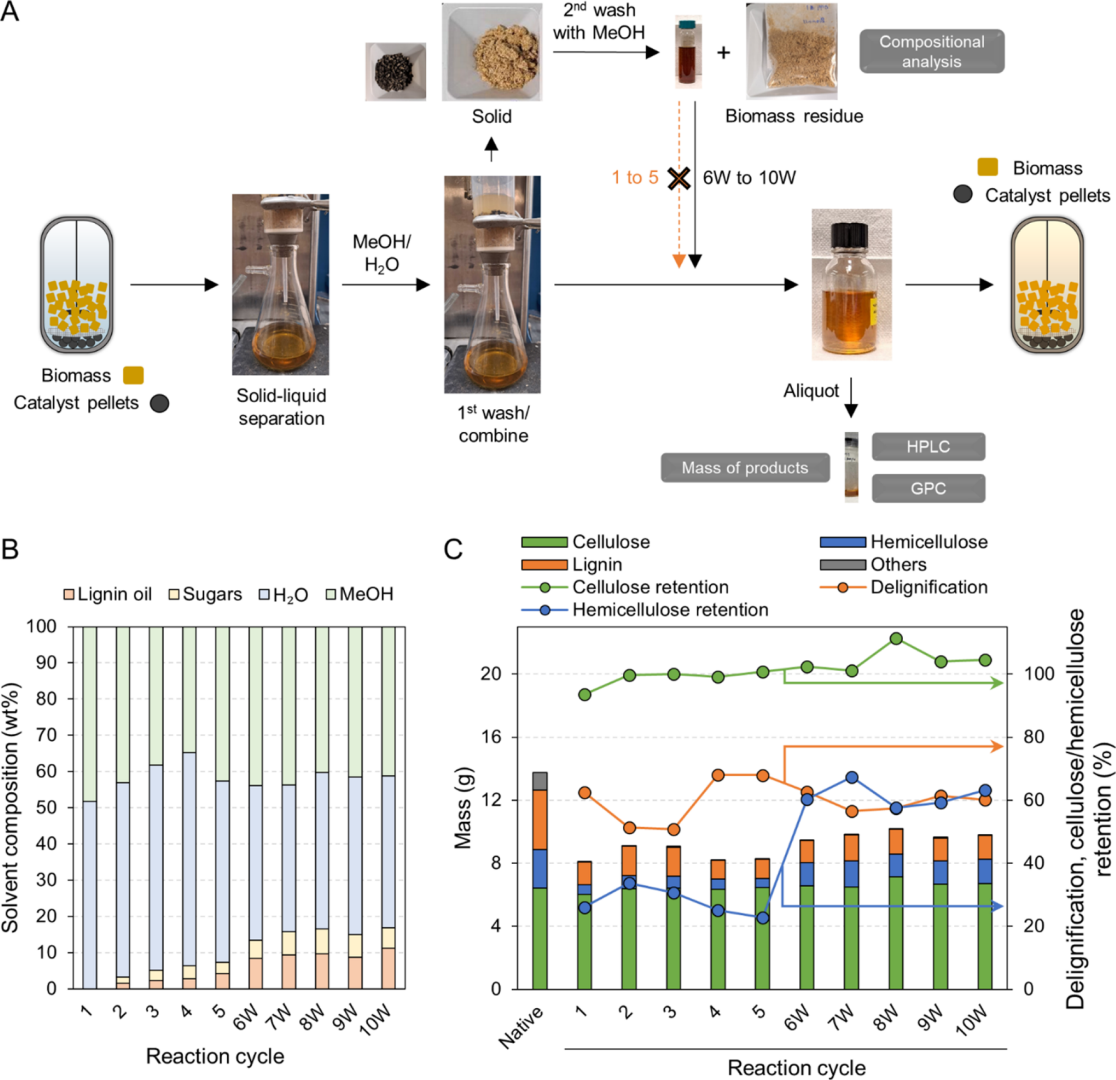
(A) The experimental scheme of subsequent fractionations and analyses of liquid and solid streams. The symbol ‘W’ denotes the inclusion of lignin oil and sugars previously dissolved in the wash solution, which was isolated by removing the washing solvent, and subsequently redissolved in the recycled reaction effluent from the preceding RCF cycle. (B) Solvent compositions for each fractionation cycle, which were derived from the reaction effluent and wash solution of the prior RCF cycle, as depicted in panel (A). To determine the concentration of solids (lignin oil + sugars) within the solvent, a sample was evaporated using a rotary evaporator, and the mass of the remaining solids was measured. The amount of lignin oil, separated from the solid residue via liquid–liquid extraction, was quantified. Any mass difference between the solid residue and the lignin oil was attributed to sugar-derived compounds. The water to methanol weight ratio was estimated using ^1^H NMR spectroscopy. (C) Compositional analysis of native and post-RCF pulp samples across fractionation cycles. Delignification and the cellulose/hemicellulose retention were calculated based on the compositional analysis data. Others include ash, extractives, acetyl, and free sugars. RCF reaction conditions: 300 mL batch reactor, 15 g of hybrid poplar, 90 g of recycled stream (solvent and wash solution), 7.5 g of 2 wt % Ru/Al_2_O_3_ catalyst pellets, 200 °C, 50 bar H_2_ (at room temperature), 2 h (after 0.5 h heating ramp). Tables S4–S5 contain the quantitative information for the data shown here.

Inspired by the promising use of lignin oil as a solvent, we conducted ten successive RCF reactions by recycling lignin oil and the solvent mixture in a 300 mL mechanically stirred batch reactor (Figure 3A). In each cycle, 15 g of hybrid poplar and 7.5 g of 2 wt % Ru/Al_2_O_3_ catalyst pellets were added while the reaction effluent was separated and recycled, maintaining the solvent-to-biomass ratio (6 g solvent/g biomass). The RCF reactions were conducted at 200 °C for 2 h. Here, we used Ru/Al_2_O_3_ catalyst pellets due to their ease of separation from biomass residue and their potential applicability in RCF processes at scale.^25^ Due to the lower Ru content (2 wt %) in the catalyst pellets compared to the powder Ru/C catalyst (5 wt %), we increased the catalyst-to-biomass ratio to maintain the Ru-to-biomass ratio. To determine the optimal residence time, we first performed RCF with a 1:1 w/w methanol–water mixture, collecting hourly samples to measure the lignin oil concentration. The concentration of lignin oil, calculated by the mass of lignin oil in each sample, plateaued after 2 h at around 2.2 wt %, as depicted in Figure S1.

Consequently, we set the reaction time at 2 h for all subsequent RCF experiments. The initial cycle was a 2 h RCF reaction with a 1:1 w/w methanol–water mixture in a 300 mL batch reactor. Compared to a 3 h RCF reaction in a 75 mL reactor (Figure 1), we observed a lower delignification extent of 63%, while the cellulose and hemicellulose retention (94 and 26%, respectively) were similar. The reduced lignin extraction extent could be attributed to the lower solvent-to-biomass ratio (6 g solvent/g biomass), compared to 9 g solvent/g biomass in the 75 mL-scale RCF reaction. After separating the liquid and solid phases, 20–30 g of methanol and water were added to the remaining solid to extract any residual lignin or sugars from biomass (1st wash in Figure 3A), standardizing the solvent mass at 90 g for the subsequent reaction. The addition of methanol and water, intended for washing, led to dilution of the lignin oil concentration to 1.5 wt %, which is lower than the 2.2 wt % achieved after a 2 h reaction without dilution, shown in Figure S1. Given that the previously reported alcohol solvent decomposition extent during RCF ranged from 0.5 to 1.4%,^11,24^ the addition of 1.5 wt % lignin oil to the solvent could replace the consumed exogenous solvent, thus enabling the reuse of the reaction effluent and the accumulation of lignin oil in the solvent.

The first five consecutive RCF reactions accumulated lignin oil, achieving an overall concentration of 4.2 wt %. The accumulation rate of lignin oil was constrained due to a portion of the extracted lignin oil remaining within the pulp residue after the first wash with methanol and water, and thus an additional wash with 30 mL of methanol was employed to recover the entrained lignin oil (2nd wash in Figure 3A). The second wash solutions collected from five cycles were then combined and subjected to methanol evaporation, yielding a lignin oil containing a solid product. This solid product, a mixture of extracted lignin and carbohydrates, was subsequently combined with the solvent recovered from the fifth cycle, elevating the lignin oil concentration to 8.4 wt % (6W in Figure 3B). Starting from the sixth RCF reaction cycle, lignin oil obtained from the second wash solution was integrated with the solvent recovered from the previous cycle to increase the accumulation rate of lignin oil. The final reaction cycle, denoted as 10W in Figure 3, used a solvent containing 11.3 wt % lignin oil, resulting from nine preceding cycles and washing steps. The quantity of sugars and polyols, calculated by mass difference between the total solid content and lignin oil dissolved in the solvent, increased over the reaction cycles. Similar to lignin oil, the concentration of sugars and polyols exhibited a rapid increase in the 6W solvent due to the inclusion of accumulated sugars and polyols from second wash solutions of the first five cycles. The methanol-to-water ratio, measured by ^1^H NMR spectroscopy, decreased over the first four cycles, likely attributed to methanol loss during RCF and vacuum-assisted separation. From the fifth cycle, adjustments were made to methanol and water volumes during washing to maintain the methanol-to-water ratio closer to 1:1 (Table S4).

Throughout the fractionation sequences, delignification levels consistently remained around 60% and cellulose retention always was 93% or higher. The cellulose retention data exceeding 100% could be because some sugars dissolved in the solvent potentially were trapped in the pulp, even after several washes. These sugars could then be inaccurately measured as cellulose during compositional analysis, leading to an overestimation of the cellulose content in the pulp. Up to the fifth fractionation cycle, hemicellulose retention varied between 22 and 34%. However, it increased to 57–68% in cycles using solvents denoted from 6W to 10W, wherein the lignin and sugars/polyols from methanol washes were incorporated into the recycled solvent. Given that a higher lignin oil concentration in the solvent enhanced hemicellulose extraction in Figure 1C, the lower extent of hemicellulose extraction observed in the sixth to 10th cycles could be due to the enriched concentration of sugars in the solvent, which could be trapped in the pulp, even after two washes, and subsequently counted as hemicellulose in the compositional analysis. Additionally, the reduced water content (40–44%) in the solvent in cycles 6W-10W, after adjusting the methanol-to-water ratio (Table S4), could also contribute to the decreased hemicellulose extraction. The aqueous fraction, obtained from liquid–liquid extraction and containing extracted and accumulated hemicellulose-derived compounds, was analyzed using HPLC. The primary products identified were 1,2-propanediol and ethylene glycol, with minor products including oligomeric xylose and xylitol, arabitol, mannitol, and glycerol (Figure S2). It is noted that no monomeric xylose and only a small quantity of oligomeric xylose were detected, indicative of the conversion of extracted hemicellulose and its reactivity during the subsequent reactions. The discrepancy in the mass of sugars and polyols can be attributed to unidentified hemicellulose-derived compounds and mass losses during liquid–liquid extraction.

### Characterization of Accumulated Lignin Oil

**Figure 4:**
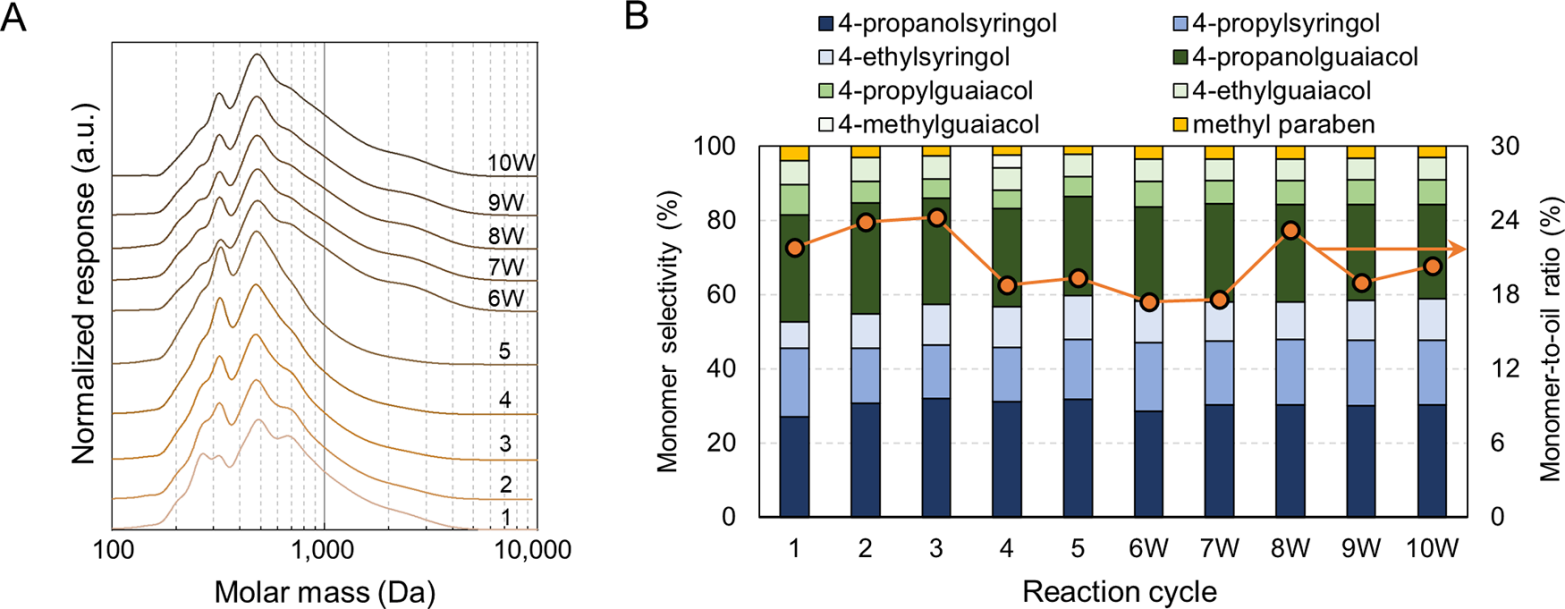
Characterization of lignin oils obtained from subsequent reactions. (A) GPC traces after acetylation and (B) monomer selectivity (left axis) and monomer-to-oil ratio (right axis). The mass of lignin oil in an aliquot was determined using the concentration of lignin oil depicted in Figure 3. The total mass of monomers was quantified by HPLC. Table S6 contains the quantitative information for the data shown here.

The lignin oil, isolated through liquid–liquid extraction of each reaction aliquot, was characterized using GPC and GC-FID. The GPC trace of lignin oil with a methanol–water mixture exhibits monomer, dimer, trimer, and tetramer peaks at 270, 320, 470, and 700 Da, respectively. The overall GPC traces remained relatively consistent throughout ten cycles. As the fractionation cycles continued, the intensity of monomer peaks slightly decreased (Figure 4A). Similarly, the monomer-to-oil ratio experienced a slight reduction of 3–4% across ten fractionation cycles (Figure 4B). Lignin oils obtained from the 6W-10W cycles showed an increased peak intensity between 2000 and 4000 Da, suggesting the presence of higher molar mass oligomers. The addition of high molar mass components occurred when lignin oils from second washing solutions were added to the solvent recovered from the fifth cycle (Figure S3). This increase may indicate that larger lignin oligomers tend to remain in the biomass and are likely liberated during the washing steps. With the methanol–water solvent, 4-propanolguaiacol and 4-propanolsyringol were the major monomers (Figure 4B). The distribution of monophenolic compounds remained similar across the reaction cycles. Together with the minimal variation in GPC traces, these observations confirm the stability of lignin oil during ten successive fractionation cycles.

### Challenges in Using Lignin Oil as a Cosolvent in RCF

As demonstrated in this work, lignin extraction efficiency (83–93%) was invariant when using solvents containing up to 80 wt % lignin oil, comparable to the conventional methanol–water solvent. RCF conducted with lignin oil as a sole solvent achieved a 67% delignification, which is similar to results of RCF using methanol as a solvent in the literature.^16,18−20^ Testing subsequent fractionation cycles, we examined the feasibility of circulating lignin oil and solvent mixtures in the RCF process. Across ten cycles, wherein the lignin oil content reached up to 11.3 wt %, the extent of delignification, GPC traces of the lignin oil, the lignin monomer distribution, and the monomer-to-lignin oil ratio all remained similar. The lack of negative impacts on delignification and lignin oil quality when using lignin oil as part of the solvent further highlights the potential for an RCF process with reduced need for exogenous organic solvents. We compared a solvent usage factor (denoted as S-factor shown in Figure S4), defined as the mass of solvent used for reaction per total mass of desired products (extracted lignin and solid carbohydrate residue). Higher concentrations of lignin oil in the solvent led to reduced S-factor values. In comparison to the solvent usage in a methanol–water RCF, solvents containing 40, 60, and 80 wt % lignin oil exhibited relative S-factors of 0.66, 0.46, and 0.26, respectively, demonstrating a significant potential for solvent reduction in RCF. Additionally, replacing light alcohols with lignin oil as a solvent would be beneficial in reducing the operating pressure, for example, 52 bar in methanol–water RCF vs 42 bar in 100 wt % lignin oil RCF, which is another major economic driver in RCF processes.

Despite these promising results, incorporating lignin oil into the solvent introduces several challenges that will require process solutions for at-scale operation. Increasing lignin in the solvent led to more lignin oil remaining in solid residue, necessitating more wash solvent to extract the retained lignin effectively. For example, the solid residue produced with 100 wt % lignin oil solvent required ten washing steps (30 mL methanol each) for thorough lignin oil extraction, which we deemed sufficient when a clear wash solution was obtained. Conversely, only two washing steps were needed when using a solvent containing 40 wt % lignin oil. Due to the additional solvent usage in the washing steps, the relative S-factor values increased when considering solvent usage for both reaction and washing steps, despite reducing solvent usage in reaction. Solvents containing 10–40 wt % lignin oil required two washing steps, resulting in S-factors of 1.4–1.6. However, RCF with 100 wt % lignin oil without methanol and water exhibited an S-factor of 7.3 solely due to ten washing steps. To reduce the demand of washing solvent and enhance the separation efficiency, flow reactor configurations for RCF with lignin-rich solvents might be beneficial, allowing for simultaneous RCF reaction, separation, and washing at or near the reaction temperature.^14,15,18,26−31^

Solvents with high lignin concentration (60–100 wt %) resulted in reduced cellulose retention extents (71–85%) while no significant cellulose extraction was detected with up to 40 wt % lignin oil concentration in the solvent. Although the reduced carbohydrate retention does not significantly impact the calculated S-factor (Figure S4), carbohydrate extraction could negatively affect the process economics because the extracted cellulose and hemicellulose could be converted to a wide slate of products, as seen with the conversion of hemicellulose (Figure S2). Therefore, identifying the products from the extracted carbohydrates and utilizing the entire slate of the carbohydrate-derived products will also need to be addressed for at-scale process feasibility. Additionally, the properties of lignin oil, influenced by the feedstock and extraction conditions, could affect the variability of delignification and polysaccharide retention, which should be investigated in future work.

## Supporting Information Available

Experimental procedures, RCF time profile, HPLC measurement, relative S-factor, GPC traces, quantitative information for the data shown in the manuscript (PDF)
