# Supplementary material for: Reducing Solvent Consumption in Reductive Catalytic Fractionation through Lignin Oil Recycling
Source: ACS Sustain Chem Eng. 2024 Aug 14;12(34):12919–26. doi: 10.1021/acssuschemeng.4c04089 (PMC11351702; doi:10.1021/acssuschemeng.4c04089)
Supplement: Supplementary file 1 — sc4c04089_si_001.pdf [file sc4c04089_si_001.pdf]

Supporting Information

Reducing solvent consumption in reductive catalytic fractionation through lignin oil recycling

Jun Hee Jang,[1,2,3] Júlia Callejón Álvarez,[1,2,4] Quinn S. Neuendorf,[1] Yuriy Román-Leshkov,[5,*] and Gregg T. Beckham[1,2,*]

1. Renewable Resources and Enabling Sciences Center, National Renewable Energy Laboratory, Golden, CO 80401, USA
2. Center for Bioenergy Innovation, Oak Ridge National Laboratory, Oak Ridge, TN 37830, USA
3. Current address: Department of Chemical Engineering, Rowan University, Glassboro, NJ 08028, USA
4. Department of Chemical and Biological Engineering, University of Colorado Boulder, Boulder, 80303, CO, USA
5. Department of Chemical Engineering, Massachusetts Institute of Technology, Cambridge, MA 02139, USA

* Correspondence: gregg.beckham@nrel.gov (G.T.B.), yroman@mit.edu (Y.R.-L.)

Table of Contents

Supplemental experimental procedures……………………………………………………………………………Page S2-4

Figure S1. Time profile of the lignin oil concentration……………………………………………………………Page S5

Figure S2. HPLC measurement of lignin, polyols, and sugars …………………………………………………Page S5

Figure S3. Supplemental GPC traces ……………………………………………………………………………Page S6

Figure S4. Relative S-factor ………………………………………………………………………………………Page S6

Tables S1-S6. quantitative information for the data shown in the manuscript…………………………Page S7-12

References………………………………………………………………………………………………………Page S13

Supplemental experimental procedures

Chemicals. All commercial materials were used as received. Ethanol (≥99.6%), methanol (≥99.8%), ethyl acetate (≥99.5%), and ruthenium on alumina pellets (2 wt%) were purchased from Fischer Scientific. Sulfuric acid (72 wt%) was purchased from Macron. Acetone-d_6 (99.9 atom% D), *n*-butanol (99.9%), pyridine (anhydrous, 99.8%), acetic anhydride (99.5%), tetrahydrofuran (inhibitor-free, ≥99.9%), dichloromethane (≥99.8%) ruthenium on carbon (Ru/C, 5 wt%) were purchased from Sigma-Aldrich.

Biomass substrate. Hybrid poplar provided by Greenwood Resources was harvested in Morrow County, OR in 2013. The biomass substrate was screened to 2 mm using knife-milling (Thomas Scientific Wiley Mill) and stored at room temperature.[1] Compositional analyses were performed based on a standard compositional analysis using NREL's Laboratory Analytical Procedure described elsewhere.[2-5] **Table S1** contains the composition of fresh hybrid poplar.

Preparation of Lignin oil. Lignin oil was prepared in a 7.6 L Parr batch reactor. The reactor was loaded with 300 g poplar, 30 g of 5 wt% Ru/C, and 3 L methanol. After placing the reactor head on the well and sealing the contents within, a pressure test was performed at 1,700 psig. The reactor was then flushed two additional times with N_2 before pressurizing at 435 psig with H_2. The temperature was set to 225°C, with a 3-hour reaction time at temperature and an additional 75 minutes of ramping to reach the set point. Mechanical stirring was used for mixing and the pressure at 225°C was 1,600 psig. To end the reaction, the heat was turned off and cooling water turned on, which was distributed throughout the reactor via a cooling loop. The reaction liquor was obtained by using a peristaltic pump with a frit attachment to separate the liquor from the biomass and catalyst. The liquor underwent methanol removal using a rotary evaporator and liquid-liquid extraction with dichloromethane and water, as described previously,[6] and Schlenk drying process.

Preparation of lignin oil containing solvent mixtures. Seven lignin oil-containing solvent mixtures (5, 10, 20, 30, 40, 60, and 80 wt% of lignin oil) were prepared by diluting the lignin oil with a 1:1 methanol/water mixture. For the experiment with 100 wt% lignin oil in **Figure 1**, the lignin oil was used as a solvent without further treatment. The total amount of each solvent mixture was 18 g.

Reductive catalytic fractionation with lignin oil as a co-solvent. Batch RCF experiments were conducted in a magnetically-stirred reactor (Parr, 5,000 series, 75 mL), as previously described.[6] Briefly, the reactor vessels were filled with 2 g of hybrid poplar, 18 g of the prepared solvent, 400 mg of 5 wt% Ru/C, and 30 bar of H_2 (**Figure 1**). Subsequently, the reactor vessels were heated to 200°C with a heating time of 30 min and maintained for 3 h at this temperature. After the reaction, the reactor vessels were quenched in an ice bath and depressurized at room temperature. The reaction slurry was filtered under vacuum using a filter funnel. The reactor and solid residue were rinsed with 30 mL of methanol. This wash solution was filtered and added to the reaction filtrate. The combined filtrate was then analyzed with high-performance liquid chromatography (HPLC) to quantify lignin-derived monomers (**Figure 2**). Regarding the solid residue, to remove residual lignin entrained in solid residue, additional washing steps (30 mL methanol each) were repeated until we obtained a clear wash solution, indicating sufficient washing. The catalyst and biomass residue were separated using water and *n*-butanol, as described by Renders *et al.*[7] The isolated biomass residue was then dried under vacuum at 40°C overnight and subjected to compositional analysis. We posited that the separation was effective based on the negligible (<0.5%) ash content (**Tables S1**) in the separated biomass residue.

Subsequent ten fractionation reductive catalytic fractionation with recycling lignin and solvent. Ten successive RCF reactions by recycling lignin oil and the solvent mixture were conducted in a 300 mL mechanically stirred batch reactor (**Figure 3**). In each cycle, 15 g of hybrid poplar and 7.5 g of 2 wt% Ru/Al_2O_3 catalyst pellets were added, while the reaction effluent was separated and recycled, maintaining the solvent-to-biomass ratio (6 g solvent/g biomass). The reaction vessel was pressurized with 50 bar of H_2 and heated to 200°C for 30 min. At the set temperature, RCF was conducted for 2 h. The initial cycle involved a 2 h RCF reaction with 90 g of a 50:50 wt/wt methanol/water mixture. After the reaction, the reaction slurry was filtered under vacuum using a filter funnel. The reactor and solid residue were rinsed with 20-30 g of methanol and water to extract any residual lignin or sugars entrained in biomass (1st wash in **Figure 3A**), standardizing the recycled solvent mass at 90 g for the subsequent reaction. This wash solution was filtered and added to the reaction filtrate. Before recycling the combined filtrate, a 1 g of aliquot of the recycled solvent was taken for further analysis. From the solid residue, catalyst pellets were separated from biomass residue. The separated biomass residue underwent an additional wash with 30 mL methanol (2nd wash in **Figure 3A**), followed by vacuum drying at 40°C, and compositional analysis. The second cycle was a 2 h RCF reaction with 90 g of the recycled stream, including solvent

and wash solution from the first cycle, by adding 15 g of fresh hybrid poplar and 7.5 g of fresh catalyst pellets. Likewise, the RCF and workup processes were repeated for 10 cycles.

Aliquot analysis. Ten aliquot samples were obtained from ten subsequent fractionation experiments. The water to methanol weight ratio was estimated using ^{1}H NMR spectroscopy. To determine the concentration of solids (lignin oil + sugars), each aliquot sample was evaporated using a rotary evaporator, and the mass of the remaining solids was measured. The amount of lignin oil, separated from the solid residue via liquid-liquid extraction with ethyl acetate and water, was quantified. The method of liquid-liquid extraction was reported elsewhere.[6] Any mass difference between the solid residue and the lignin oil was attributed to sugar-derived compounds. The isolated lignin oil was then subjected to HPLC analysis for monomer quantification and gel permeation chromatography (GPC) analysis for investigating the molar mass distribution of the lignin oil.

High-performance liquid chromatography (HPLC) analysis. Lignin-derived monomers were quantified using an Agilent 1290 series equipped with a diode array detector (DAD). The detailed methods were described in our previous study.[8] Briefly, the reaction samples were diluted with methanol to make the lignin monomer concentration is in the range of calibration curves. Samples and standards (1.0 µL) were injected into a Phenomenex Luna C18(2)-HST column at 35 °C. Separation was conducted using mobile phases of 0.2% formic acid in water (A) and acetonitrile (B), at 0.5 mL/min flow. The gradient began with 95% A and 5% B, shifting to 72% A and 28% B by 2.4 min, then to 40% A and 60% B by 4.0 min, and returning to initial conditions after 6.5 min, with a total run time of 8 min. Monitored analytes included guaiacol derivatives, isoeugenol, syringol derivatives, phenol, methyl paraben, and various acids, at a detection wavelength of 280 nm, with a quantitation range of 1-500 µg/mL and an R2 $\geq$ 0.995. The monomer selectivity was calculated on a weight basis using the following equation:

$$Monomer \text{ selectivity } (\%) = \frac{mass_{monomer\ i}}{mass_{total\ detected\ monomers}} \times 100 \ (\mathbf{S1})$$

Gel permeation chromatography (GPC). Lignin oil molar mass distributions of the isolated lignin oil obtained through ten successive fractionation cycles were determined using GPC as described previously.[1] Briefly, around 20 mg of oil was acetylated using a 1 mL acetic anhydride-pyridine mix (1:1 v/v) at 40°C for 24 h with stirring. The reaction was quenched with 1 mL methanol, then evaporated under nitrogen. This methanol addition and evaporation process was repeated five times. The acetylated samples were vacuum-dried at 40°C overnight, dissolved in tetrahydrofuran, and filtered through a 0.2 µm filter. For GPC analysis, 20 µL of each sample was analyzed using HPLC with PLgel columns (7.5 x 300 mm, 10 µm pore size in 50 Å, 103 Å, and 104 Å) by Agilent Technologies, at room temperature with tetrahydrofuran flow (1 mL/min) for 40 minutes. Detection was performed at 210 nm, 260 nm, and 270 nm. Polystyrene standards were used for calibration.

Proton (^{1}H) NMR spectroscopy. To prepare an NMR sample, 20 mg of the aliquot sample from ten fractionation cycles was dissolved in 700 µL acetone-d_6. NMR spectroscopy was conducted using a Bruker Avance III 400 MHz with a 5 mm Prodigy BBO LN2 cryoprobe.

Biomass residue characterization. Biomass residues from RCF were subjected to a standard compositional analysis protocol.[2-5] Briefly, the biomass residue, separated from catalysts, was dried and then hydrolyzed using 72% sulfuric acid at 30°C for 1 hour. This mixture was diluted to 4% acid concentration, autoclaved at 121°C for 1 hour, and filtered to obtain Klason lignin. Acid-soluble lignin levels were measured by their absorbance at 240 nm. Carbohydrates were quantified through HPLC, using a Shodex Sugar SP0810 column and a de-ashing guard column. Polymeric sugars like glucan, xylan, galactan, arabinan, and mannan were estimated from their monomeric forms, with glucan calculated from glucose in cellulose and hemicellulose. Delignification and retention of glucan/xylan were calculated using the following equations:

$$Delignification\ (\%) = \left(1 - \frac{mass_{lignin\ in\ biomass\ residue}}{mass_{initial\ lignin\ in\ fresh\ biomass}}\right) \times 100\ (\mathbf{S2})$$

$$Glucan\ retention\ (\%) = \left(\frac{mass_{glucan\ in\ biomass\ residue}}{mass_{initial\ glucan\ in\ fresh\ biomass}}\right) \times 100\ (\mathbf{S3})$$

$$Xylan\ retention\ (\%) = \left(\frac{mass_{xylan\ in\ biomass\ residue}}{mass_{initial\ xylan\ in\ fresh\ biomass}}\right) \times 100\ (\mathbf{S4})$$

Quantification of acetic acid and methyl acetate. For the quantification of acetic acid and methyl acetate, the lignin oil sample was diluted in methanol solvent to a concentration of 20 mg of oil /mL of methanol, while post reaction samples were obtained directly from the reaction liquor in methanol/water and were not diluted further.

Acetic acid was quantified using an Agilent 1260 Infinity II LC System with a refractive index detection (RID). Agilent OpenLab CDS, ChemStation software version A.01.11.138 was used to collect and quantitate the analyte of interest. The analyte of interest was separated on a Rezex ROA-Organic Acid H+ (8%) 50 x 7.8 mm column (phenomenex) with an isocratic flow of 0.02 N sulfuric acid (0.01 M) at 0.5 mL/min for a total run time of 30 minutes. Acetic acid standard and samples were injected into the column at a volume of 20 µL, while the temperature of the column was maintained at 70°C and the detector at 55°C. A minimum of 9 calibration levels was used to quantitate acetic acid between 25 µg/mL to 5000 µg/mL with an r^2 coefficient of 0.995 or better.

Quantification of methyl acetate was performed on an Agilent 7890A Gas Chomatograph (GC) equipped with a 5975C Mass Spectrometer Detector (MS). Agilent MSD Productivity Chemstation G1701 software version E.02.02.1431 was used to collect and quantitate the analyte of interest. Samples were injected at a volume of 1 uL into the GC/MS in split mode. The analyte of interest was separated on an Agilent DB-624 UI column (Part # 122-1334UI). The GC/MS method consisted of a front inlet temperature of 250°C with a split ratio set to 20:1, and an auxiliary transfer line temperature of 250°C. A constant flow of 1 ml/min was held throughout the run. Starting temperature of 70°C was held for 1 min and then ramped at 20°C/min to a temperature of 260°C and held for 1.5 min. The method resulted in a run time of 12 min for each sample. Sample TICs (Total Ion Count) were collected on the MS system from 10 m/z to 550 m/z. A minimum of 5 calibration levels was used to quantitate methyl acetate between 1 µg/mL to 750 µg/mL with an r^2 coefficient of 0.995 or better. A check calibration standard (CCS) was analyzed every 10 samples to ensure the integrity of the initial calibration.

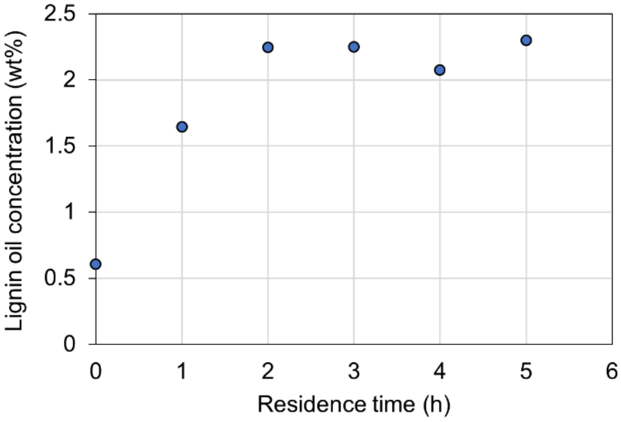

Figure S1. Time profile of the lignin oil concentration. Each time sample was subjected to solvent evaporation and liquid-liquid extraction to calculate the concentration of lignin oil. RCF reaction conditions: 300 mL batch reactor, 15 g of hybrid poplar, 90 g of solvent, 7.5 g of 2 wt% Ru/Al_2O_3 catalyst pellets, 200°C, 50 bar H_2 (at room temperature).

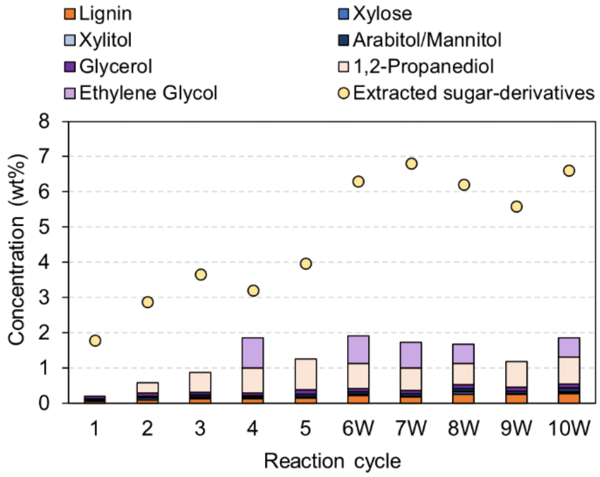

Figure S2. HPLC measurement of lignin, polyols, and sugars was performed on the aqueous phase after liquid-liquid extraction of aliquot samples from subsequent RCF reactions. The amount of extracted sugar derivatives was calculated by the difference between 1) the total solid residue in the aliquot sample measured after solvent drying and 2) the lignin oil measured in the organic phase after liquid-liquid extraction of aliquot samples.

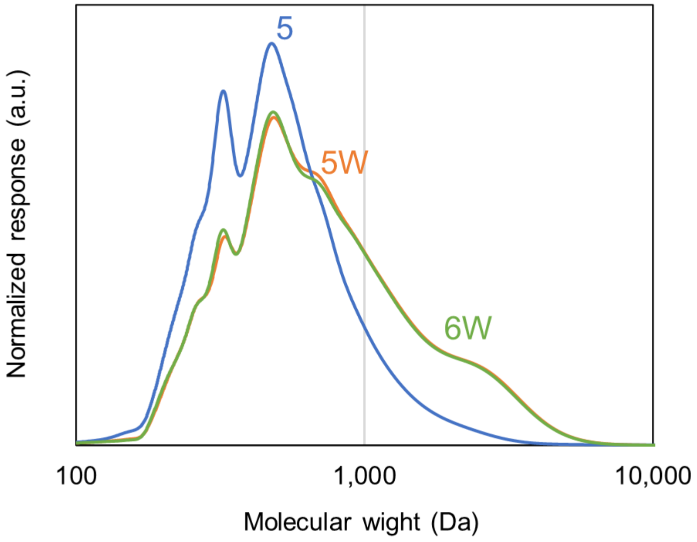

Figure S3. GPC traces after acetylation of samples of 5, 5W, and 6W. Sample 5 represents the solvent recovered from the fifth cycle (reaction and 1st wash). Lignin oil from the 2nd wash solutions of the first five cycles was added to sample 5, resulting in sample 5W. The sixth cycle used sample 5W as a solvent and post RCF and subsequent the 1st and 2nd washes yielded sample 6W.

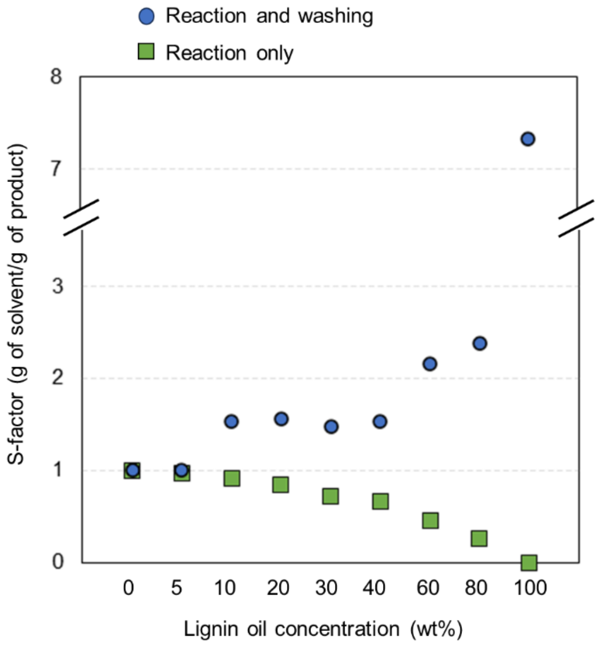

Figure S4. The relative S-factor based on the S-factor value from RCF with methanol/water. The S-factor represents the mass of solvent (methanol and water) used for the reaction (and washing) per total mass of the desired RCF products, which include extracted lignin and solid carbohydrate residues.

Table S1. Compositional analysis of native and biomass residue samples from RCF with varied solvent compositions. **Table S1** is related to **Figure 1C**.

Sample		Cellulose (g)	Hemicellulose[a] (g)	Lignin (g)	Others[b] (g) Ash	Extractives	Acetates	Total[c] (g)	Delignification[d] (%)	Cellulose retention rate[e] (%)	Hemicellulose retention rate[f] (%)
Hybrid poplar		0.856	0.327	0.503	0.01	0.07	0.07	1.836	-	-	-
Post-RCF sample	0 wt%	0.87	0.087	0.083	-	-	-	1.047	83.4	101.7	26.7
	5 wt%	0.857	0.065	0.083	-	-	-	1.012	83.5	100.1	19.9
	10 wt%	0.862	0.074	0.072	-	-	-	1.014	85.8	100.8	22.5
	20 wt%	0.818	0.052	0.066	-	-	-	0.944	86.8	95.6	15.9
	30 wt%	0.852	0.046	0.056	-	-	0.01	0.962	88.9	99.6	14.0
	40 wt%	0.819	0.02	0.085	-	-	-	0.931	83.2	95.7	6.1
	60 wt%	0.726	0.008	0.035	-	-	-	0.778	93.0	84.8	2.6
	80 wt%	0.608	0.006	0.077	-	-	-	0.699	84.6	71.1	1.9
	100 wt%	0.702	0.033	0.167	-	-	0.01	0.915	66.7	82.0	10.1

[a]Xylan + galactan + arabinan + mannan. [b]Ash + extractives + acetates. [c]Mass of the sample after drying in a 120°C overnight. [d]Calculated using **Equation S2**. [e]Calculated using **Equation S3**. [f]Calculated using **Equation S4.**

Table S2. Concentration of acetic acid and methyl acetate in the prepared lignin oil and post-RCF reaction effluent.

Entry	Acetic acid (ppm)	Methyl acetate (ppm)
Lignin oil	0	0
Post RCF reaction effluent, 0 wt%	1098	1338
Post RCF reaction effluent, 60 wt%	721	1917

Table S3. Monomer selectivity of the as-prepared lignin oil (labelled 'Lignin oil') and lignin oils obtained post-RCF. **Table S3** is related to **Figure 2**.

Sample	4-propanol syringol (%)	4-propyl syringol (%)	4-propenyl syringol (%)	4-propanol guaiacol (%)	4-propyl guaiacol (%)	4-propenyl guaiacol (%)	4-ethyl guaiacol (%)	methyl paraben (%)
Lignin oil	12.1	39.4	4.0	10.6	23.7	2.6	0.3	7.4
0 wt%	39.3	15.8	0.0	34.3	7.6	0.0	2.9	0.0
5 wt%	19.5	36.6	0.0	17.9	19.7	0.0	1.3	5.0
10 wt%	16.5	38.6	0.0	15.3	22.0	0.0	1.3	6.2
20 wt%	13.5	41.4	0.0	12.8	24.4	0.0	1.0	6.9
30 wt%	13.2	42.0	0.0	12.4	24.1	0.0	1.2	7.1
40 wt%	12.6	42.0	0.0	11.8	25.0	0.0	1.3	7.3
60 wt%	12.1	42.5	0.0	11.0	25.8	0.0	1.0	7.6
80 wt%	11.7	42.9	0.0	10.5	26.3	0.0	0.8	7.8
100 wt%	11.2	43.6	0.0	9.8	26.8	0.0	0.6	8.1

Table S4. Solvent compositions for each fractionation cycle, which were derived from the reaction effluent and wash solution of the prior RCF cycle. To determine the concentration of solids (lignin oil + sugars) within the solvent, a sample was evaporated using a rotary evaporator, and the mass of the remaining solids was measured. The amount of lignin oil, separated from the solid residue via liquid-liquid extraction, was quantified. Any mass difference between the solid residue and the lignin oil was attributed to sugar-derived compounds. The water to methanol weight ratio was estimated using ^{1}H NMR. **Table S4** is related to **Figure 3B**.

Sample	Lignin oil (%)	Sugars/polyols (%)	Water (%)	Methanol (%)	Methanol-to-water ratio
1	0	0	51.7	48.3	0.93
2	1.5	1.8	53.5	43.2	0.81
3	2.3	2.9	56.6	38.2	0.68
4	2.8	3.7	58.8	34.8	0.59
5	4.2	3.2	49.9	42.7	0.85
6W	8.4	5.1	42.6	43.9	1.03
7W	9.4	6.3	40.6	43.7	1.08
8W	9.7	6.8	43.2	40.3	0.93
9W	8.8	6.2	43.5	41.5	0.95
10W	11.3	5.6	42.0	41.2	0.98

Table S5. Compositional analysis of native and pulp samples from RCF with varied solvent compositions. **Table S5** is related to **Figure 3C**.

Sample		Cellulose (g)	Hemicellulose[a] (g)	Lignin (g)	Others[b] (g)			Total[c] (g)	Delignification[d] (%)	Cellulose retention rate[e] (%)	Hemicellulose retention rate[f] (%)
					Ash	Extractives	Acetates				
Hybrid poplar		6.42	2.45	3.77	0.09	0.49	0.50	13.77	-	-	-
Post-RCF sample	1	6.01	0.64	1.42	-	-	0.03	8.10	62.5	93.7	26.1
	2	6.4	0.83	1.84	0.03	-	0.02	9.11	51.3	99.7	33.7
	3	6.42	0.75	1.85	0.02	-	0.02	9.07	50.8	100.0	30.6
	4	6.36	0.62	1.21	-	-	0.01	8.19	68.0	99.1	25.1
	5	6.47	0.56	1.21	0.02	-	0.01	8.27	68.0	100.8	22.7
	6W	6.57	1.48	1.41	0.02	-	0.01	9.49	62.6	102.3	60.3
	7W	6.49	1.65	1.64	0.02	-	0.02	9.83	56.6	101.2	67.3
	8W	7.15	1.41	1.60	0.02	-	0.02	10.20	57.5	111.4	57.7
	9W	6.68	1.45	1.46	0.04	-	0.02	9.64	61.4	104.1	59.3
	10W	6.71	1.55	1.50	-	-	0.02	9.78	60.1	104.5	63.2

[a]Xylan + galactan + arabinan + mannan. [b]Ash + extractives + acetates. [c]Mass of the sample after drying in a 120°C overnight. [d]Calculated using **Equation S2**. [e]Calculated using **Equation S3**. [f]Calculated using **Equation S4.**

Table S6. Monomer selectivity of lignin oils obtained from subsequent reactions. **Table S6** is related to **Figure 4B**.

Sample	4-propanol syringol (%)	4-propyl syringol (%)	4-ethyl syringol (%)	4-propanol guaiacol (%)	4-propyl guaiacol (%)	4-ethyl guaiacol (%)	4-methyl guaiacol (%)	methyl paraben (%)
1	27.2	18.3	7.1	29.0	8.1	6.5	0.0	3.9
2	30.7	14.9	9.2	29.9	5.9	6.4	0.0	3.1
3	32.1	14.4	11.0	28.5	5.1	6.3	0.0	2.6
4	31.1	14.7	10.9	26.5	5.0	6.1	3.4	2.3
5	31.8	16.2	11.7	26.7	5.3	6.0	0.0	2.2
6W	28.5	18.5	11.3	25.3	6.9	6.0	0.0	3.4
7W	30.3	17.3	10.5	26.4	6.4	5.7	0.0	3.4
8W	30.4	17.5	10.2	26.2	6.6	5.8	0.0	3.4
9W	30.1	17.7	10.8	25.7	6.7	5.9	0.0	3.1
10W	30.2	17.6	11.2	25.4	6.6	6.0	0.0	3.0

References

(1) Thornburg, N. E.; Pecha, M. B.; Brandner, D. G.; Reed, M. L.; Vermaas, J. V.; Michener, W. E.; Katahira, R.; Vinzant, T. B.; Foust, T. D.; Donohoe, B. S.; et al. Mesoscale reaction-diffusion phenomena governing lignin-first biomass fractionation. *ChemSusChem* **2020**, *13* (17), 4495-4509.

(2) Sluiter, A.; Ruiz, R.; Scarlata, C.; Sluiter, J.; Templeton, D. *Determination of extractives in biomass* National Renewable Energy Laboratory, Golden, Colorado, USA, 2005.

(3) Sluiter, A.; Hames, B.; Ruiz, R.; Scarlata, C.; Sluiter, J.; Templeton, D. *Determination of sugars, byproducts, and degradation products in liquid fraction process samples* National Renewable Energy Laboratory, Golden, Colorado, USA, 2006.

(4) Sluiter, A.; Hames, B.; Ruiz, R.; Scarlata, C.; Sluiter, J.; Templeton, D. *Determination of ash in biomass*; National Renewable Energy Laboratory, Golden, Colorado, USA, 2008.

(5) Sluiter, A.; Hames, B.; Ruiz, R.; Scarlata, C.; Sluiter, J.; Templeton, D.; Crocker, D. *Determination of structural carbohydrates and lignin in biomass*; National Renewable Energy Laboratory, Golden, Colorado, USA, 2008.

(6) Jang, J. H.; Morais, A. R. C.; Browning, M.; Brandner, D. G.; Kenny, J. K.; Stanley, L. M.; Happs, R. M.; Kovvali, A. S.; Cutler, J. I.; Román-Leshkov, Y.; et al. Feedstock-agnostic reductive catalytic fractionation in alcohol and alcohol–water mixtures. *Green Chem.* **2023**, *25* (9), 3660-3670.

(7) Renders, T.; Cooreman, E.; Van den Bosch, S.; Schutyser, W.; Koelewijn, S. F.; Vangeel, T.; Deneyer, A.; Van den Bossche, G.; Courtin, C. M.; Sels, B. F. Catalytic lignocellulose biorefining in n-butanol/water: a one-pot approach toward phenolics, polyols, and cellulose. *Green Chem.* **2018**, *20* (20), 4607-4619.

(8) Choi, H.; Alherech, M.; Jang, J.; Woodworth, S. P.; Ramirez, K. J.; Karp, E. M.; Beckham, G. T. Counter-current chromatography for lignin monomer-monomer and monomer-oligomer separations from reductive catalytic fractionation oil. *Accepted in Green Chem.* **2024**.
